# Impairment of small somatic and autonomic nerve fibres in intensive care unit patients with severe sepsis and critical illness polyneuropathy – a single center controlled observational study

**DOI:** 10.1186/1471-2377-13-159

**Published:** 2013-11-01

**Authors:** Hubertus Axer, Alexander Grimm, Christine Porzelius, Ulrike Teschner, Ulrike Schumacher, Otto W Witte, Frank M Brunkhorst

**Affiliations:** 1 Hans Berger Department of Neurology, Jena University Hospital, Friedrich-Schiller-University Jena, Erlanger Allee 101, D-07747 Jena, Germany; 2 Center for Sepsis Control and Care (CSCC), Jena University Hospital, Jena, Germany; 3 Institute for Medical Informatics, Statistics and Epidemiology (IMISE), University Leipzig, Leipzig, Germany; 4 Center for Clinical Studies (CCS), Jena University Hospital, Jena, Germany; 5 Paul-Martini-Clinical Sepsis Research Unit, Department of Anaesthesiology and Intensive Care Medicine, Jena University Hospital, Jena, Germany

**Keywords:** Critical illness polyneuropathy, CIP, Severe sepsis, Skin biopsies, Small fibre neuropathy

## Abstract

**Background:**

Axonal damage in large myelinated nerve fibres occurs in about 70% of patients with severe sepsis, known as critical illness polyneuropathy and contributes significantly to an increased short- and long-term morbidity and mortality in this population. Among other pathophysiological mechanisms, autonomic dysregulation, characterized by high concentrations of circulating catecholamines in the presence of impaired sympathetic modulation of heart and vessels have been discussed. We hypothesize that autonomic small fibre neuropathy play an important role in autonomic failure.

**Methods/Design:**

Single center, non-randomized, controlled, observational study. Skin biopsies of patients with severe sepsis and/or septic shock are compared with those of age-matched controls. In order to assess impairment of small nerve fibres, skin biopsies are taken at onset of severe sepsis, and two and 16 weeks later. Intraepidermal nerve fibre densities are histologically analyzed using anti protein gene product (PGP) 9.5 immunostaining. In addition, standardized clinical examinations, as Medical Research Council (MRC) scores of muscle strength, Rankin scores, and standardized nerve conduction studies of the right median nerve, the right tibial nerve, the left fibular nerve, and both sural nerves are performed, to identify critical illness polyneuropathy and to neurophysiologically quantify the damage of large nerve fibres.

**Discussion:**

The study will allow to describe the frequency of small fibre neuropathy in patients with severe sepsis up to four months after onset of severe sepsis and to evaluate its relationship to critical illness polyneuropathy.

**Trial registration:**

The trial has been registered to the German Clinical Trials Register. The trial registration number is DRKS-ID: DRKS00000642.

## Background

Severe sepsis and septic shock affect hospitals and health care systems worldwide and represent the third leading cause of death in industrialized countries [1]. About 70% of patients develop critical illness polyneuropathy (CIP) [2,3], the frequency increases up to 100% in patients with multi-organ failure [4,5].

Critical illness polyneuropathy (CIP) and myopathy (CIM) share the major clinical sign of symmetric and flaccid weakness of muscles and the absence of deep tendon reflexes [6-9]. Patients with CIP also show a distal loss of sensitivity to pain, temperature, and vibration. Electromyography (EMG) and nerve conduction studies are the electrophysiological gold standards to confirm CIP or CIM. Typical signs are reductions in amplitudes of compound muscle action potentials and/or sensory nerve action potentials in contrast to preserved nerve conduction velocity indicating axonal damage [10]. CIP and CIM severely impair rehabilitation and lead to prolonged weaning times from the respirator [11].

In addition, autonomic dysregulation plays a significant role in the acute phase of severe sepsis [12], since it is characterized by high concentrations of circulating catecholamines in the presence of impaired sympathetic modulation of heart and vessels, thus contributing to circulatory failure in severe sepsis. Even though autonomic dysregulation is associated with increased long-term mortality rates [13,14], the role of an impairment of small peripheral nerve fibres has never been investigated. Whereas CIP is quite well studied using electrophysiological measurements as electromyography and nerve conduction studies, little information is available about the impairment of small nerve fibres (C-fibres) and autonomic nerve fibres in the course of the disease. Nerve conduction studies are able to measure thick myelinated nerve fibres only, because these mainly compose compound muscle action potentials and sensory nerve action potentials. Histopathological data originate solely from nerve and muscle biopsies [15,16].

Recently, novel methods using skin biopsies have added a new tool for providing diagnostic information on small nerve fibres. Skin biopsies are safe, minimally invasive and painless [17] and can be performed at every site of the body using a disposable 3 mm diameter punch and usually no suture is required. Its minimal invasiveness makes skin biopsy a useful tool in clinical practice for diagnosing and monitoring the progression of peripheral nerve damage. Intraepidermal nerve fibres (IENFs) can be labelled with antibodies against protein gene product (PGP) 9.5 [18,19]. The innervation of dermal autonomic structures (sweat glands, blood vessels, hair follicles) can be detected using antibodies against adrenergic, noradrenergic, cholinergic, and vasodilatatory peptidergic fibres [20]. Therefore, skin biopsy is able to detect separately and specifically small somatic and autonomic nerve fibres. Histological measurements are highly reliable [21] and correlate significantly to stereologically estimated nerve fibre densities [22]. Intraepidermal nerve fibre density is a quantitative estimate of the number of nerve fibres in the histologic sample measured in nerve fibres per millimeter. Normative values of intraepidermal nerve fibre density in healthy populations already exist [23-25]. Quantification of IENF density closely correlates with clinical symptoms and is more sensitive than sensory nerve conduction studies and sural nerve biopsies in diagnosing small fibre neuropathy. Diagnostic efficiency and predictive values of this technique are very high (level A recommendation) [18,19].

Although pathophysiological mechanisms of CIP development have been described in the past, many aspects remain unclear [4,9]. Former studies mainly have focused on large, thick myelinated nerve fibres, because these contribute to muscle weakness. Impairment of small nerve fibres and autonomic nerve fibres and their role in autonomic failure is less understood.

## Methods/Design

### Study design

Single center, non-randomized, controlled, observational study.

### Ethical and research governance approval

The study has been granted ethical approval from the Ethics Committee of the Friedrich Schiller University Jena (No. 2771-02/10). Written informed consent is obtained from all patients or their legal representatives. The study is funded by the German Ministry of Education and Research (BMBF), Grant No 01 E0 1002.

### Trial duration

The study started in October 2010 and will end in August 2015. Recruitment of patients started on February 2011 and will proceed until April 2015.

### Participants

200 patients with severe sepsis and/or septic shock and 60 age-matched controls. Patients are screened daily for eligibility by four trained ICU research nurses in one medical, two surgical and one neurological ICUs, comprising a total of 70 beds in Jena University Hospital.

### Inclusion and exclusion criteria

Patients with severe sepsis and/or septic shock according to published criteria [26,27], age ≥ 18 years, severe sepsis and/or septic shock onset ≥ 4 days, written informed consent obtained from patients or their legal representatives. Exclusion criteria are history of neuromuscular disorders, such as polyneuropathy, myasthenia gravis, myopathy and others), known alcohol abuse, high-dose steroid therapy before sepsis (≥16 mg/kg body weight for 5 days), ICU stay ≥8 days, participation in another clinical study, platelet count < 40 Gpt/l, PTT > 60 s, INR > 1.7, likely to die within less than 24 hours.

Inclusion criteria for the control group are written informed consent. Exclusion criteria are history of neuromuscular disorders, known alcohol abuse, high-dose steroid therapy, platelet count < 40 Gpt/l, PTT > 60 s, INR > 1.7.

### Study visits and interventions

#### *Severe sepsis/septic shock group*

Study visits are performed between days 2–5 after severe sepsis/septic shock onset, and on day 14 and 4 months later (Table 1).

**Table 1:** Visits and diagnostic procedures

**Visit**	**Time point**	**Diagnostic procedures**
1	In the first week after onset of acute sepsis	1st skin biopsy at thigh and ankle, neurological clinical examination (MRC score of muscle strength, ONLS, Rankin score), nerve conduction studies, sympathetic skin response
2	2 weeks later	2nd skin biopsy at thigh and ankle, neurological clinical examination, nerve conduction studies, sympathetic skin response
3	4 months later	3d skin biopsy at thigh and ankle, neurological clinical examination, nerve conduction studies, sympathetic skin response

**Neurological examinations** Patients are undergoing a standardized clinical neurological examination including evaluation of MRC (Medical Research Council) muscle strength, status of muscle reflexes, Rankin score, and ONLS (Overall Neuropathy Limitations Scale) [28].

**Nerve conduction studies** Standardized nerve conduction studies are carried out at each visit using a portable electrophysiologic device. The right median nerve, the right tibial nerve, the left fibular nerve, and both sural nerves are measured. Motor and sensory nerve responses are assessed for the right median nerve, motor responses only for the tibial and fibular nerve, and sensory nerve responses for both sural nerves. Sympathetic skin responses are measured at hands and feet as a neurophysiological equivalent of autonomic function.

**Skin biopsies** Skin biopsies are collected from ankle and thigh at each visit using a 3 mm biopsy punch. Bright field microscopy for routine diagnostic purposes is used to analyse skin biopsies. The biopsied tissue is cut into 50 μm thick sections and immunostained with anti-PGP 9.5 antibodies to label small nerve fibres in the skin. Intraepidermal nerve fibre density is calculated by the the number of stained nerve fibres crossing the basal lamina of the skin divided by the length of the epidermal surface.

#### *Control group*

Controls are visited once and get a clinical neurological examination and skin biopsies taken from ankle and thigh.

### Primary endpoint

Frequency of small fibre neuropathy, characterized as density of intraepidermal nerve fibers (IENF) derived from skin biopsies, two weeks after onset of severe sepsis/septic shock patients in comparison to controls.

### Secondary endpoints

• Frequency of small fibre neuropathy, characterized as density of intraepidermal nerve fibers (IENF) derived from skin biopsies, two to five days after onset of severe sepsis/septic shock in patients who developed CIP in comparison to patients without CIP development.

• Frequency of small fibre neuropathy, characterized as density of intraepidermal nerve fibers (IENF) derived from skin biopsies, 4 months days after onset of severe sepsis/septic shock in patients who developed CIP in comparison to patients without CIP development.

• IENF density in comparison to electrophysiological measurements, clinical parameters (MRC, ONLS) at severe sepsis/shock onset, at 2 weeks and at 4 months.

### Statistical analysis

No data is available about the existence of small fibre neuropathy in severe sepsis/septic shock patients. Normative value for mean intraepidermal nerve fibre density at the ankle is supposed to be 12.4 with a standard deviation of 4.6 [24]. About 60 of 200 patients with severe sepsis/septic shock may not develop CIP (30%) in contrast to 140 (70%) patients with CIP [2]. With a control group of 60 healthy subjects, a difference of intraepidermal nerve fibre density of 2 can be detected in an unpaired *t*-test with a two-sided significance level of 5% and a power of 80%. Analysis of the primary endpoint will be done using the unpaired *t*-test. Analysis of the secondary endpoints will be done using descriptive statistics and unpaired t-tests, where appropriate, which will be mainly hypothesis-generating. If applicable, additional analysis using multivariate regression models will be done. Due to the explorative character of the secondary analyses, no α correction will be performed.

## Discussion

This is to our knowledge the first study to address the frequency of small fibre neuropathy in patients with severe sepsis/septic shock, treated in intensive care units. Skin biopsies have become a valuable tool in the evaluation of patients with neuropathy [19] with good evidence of reliable normative data [25]. Detection of an impairment of small nerve fibers in sepsis may give further insights into the pathophysiological role of autonomic neuropathy in patients with severe sepsis/septic shock [12].

Preliminary experiences from our ongoing study suggest that difficulties in patient recruitment may arise because of bleeding risks as a contraindication for skin biopsies. Furthermore, gaining informed consent from the legal representative of severe sepsis/septic shock patients is difficult to obtain in the acute care setting and may limit patient recruitment. Moreover, first results of the prospective severe sepsis/septic shock registry of the Centre of Sepsis Control and Care (CSCC) at the study center Jena University Hospital revealed higher mortality rates as suspected [29], so that relatively few patients may be available for follow-up examinations. Finally, the drop-out rate at 4 months is high due patient’s preferences and decisions. This is highly important for planning further studies on long-term sequelae in severe sepsis/septic shock patients.

### Trial status

Recruitment of patients started on February 2011 and will proceed until April 2015. Currently (July 2013), 40 patients with severe sepsis and/or septic shock and 40 controls have been recruited.

## Abbreviations

CIP: Critical illness polyneuropathy; CIM: Critical illness myopathy; EMG: Electromyography; ICU: Intensive care unit; IENF: Intraepidermal nerve fibre; INR: International Normalized Ratio; MRC: Medical Research Council; ONLS: Overall neuropathy limitations scale; PTT: Partial thromboplastin time.

## Competing interests

The authors declare that they have no competing interests.

## Authors’ contributions

HA, FMB, and OW developed the project. HA obtained funding. All authors participated in the final design of the study. HA wrote the first draft of this paper and the other authors provided input. AG and UT are involved in data collection. HA supervises the project. CP and US are the study statisticians. CP, HA, US, and FMB will analyze the data. All authors read and approved the final manuscript.

## Pre-publication history

The pre-publication history for this paper can be accessed here:

http://www.biomedcentral.com/1471-2377/13/159/prepub
